# Live and Heat-Killed* Lactobacillus rhamnosus* ATCC 7469 May Induce Modulatory Cytokines Profiles on Macrophages RAW 264.7

**DOI:** 10.1155/2015/716749

**Published:** 2015-11-16

**Authors:** Adeline Lacerda Jorjão, Felipe Eduardo de Oliveira, Mariella Vieira Pereira Leão, Cláudio Antonio Talge Carvalho, Antonio Olavo Cardoso Jorge, Luciane Dias de Oliveira

**Affiliations:** ^1^Department of Biosciences and Oral Diagnosis, Laboratory of Microbiology and Immunology, Institute of Science and Technology, Universidade Estadual Paulista (UNESP), Avenida Engenheiro Francisco José Longo, 777 Jardim São Dimas, 12245-000 São José dos Campos, SP, Brazil; ^2^Bioscience Basic Institute, University of Taubaté, Street Expedicionário Ernesto Pereira 3, 12020-130 Taubaté, SP, Brazil; ^3^Department of Restorative Dentistry, Institute of Science and Technology, Universidade Estadual Paulista (UNESP), Avenida Engenheiro Francisco José Longo, 777 Jardim São Dimas, 12245-000 São José dos Campos, SP, Brazil

## Abstract

This study aimed to evaluate the capacity of* Lactobacillus rhamnosus* and/or its products to induce the synthesis of cytokines (TNF-*α*, IL-1*β*, IL-4, IL-6, IL-10, and IL-12) by mouse macrophages (RAW 264.7). Three microorganism preparations were used: live* L*.* rhamnosus* (LLR) suspension, heat-killed* L*.* rhamnosus* (HKLR) suspension, and the supernatant of a heat-killed* L*.* rhamnosus* (SHKLR) suspension, which were cultured with macrophages (37°C, 5% CO_2_) for 2 h and 30 min. After that, cells were cultured for 16 h. The supernatants were used for the quantitation of cytokines, by ELISA. The results were compared with the synthesis induced by lipopolysaccharide (LPS) and analysed, using ANOVA and Tukey test, 5%. LLR and HKLR groups were able to significantly increase the production of TNF-*α*, IL-6, and IL-10 (*P* < 0.05). SHKLR also significantly increased the production of TNF-*α* and IL-10 (*P* < 0.05) but not IL-6 (*P* > 0.05). All the* L*.* rhamnosus* suspensions were not able to produce detectable levels of IL-1*β* or significant levels of IL-4 and IL-12 (*P* > 0.05). In conclusion, live and heat-killed* L*.* rhamnosus* suspensions were able to induce the synthesis of different cytokines with proinflammatory (TNF-*α* and IL-6) or regulatory (IL-10) functions, suggesting the role of strain* L*.* rhamnosus* ATCC 7469 in the modulation or in the stimulation of immune responses.

## 1. Introduction

According to WHO [1], probiotics are “live organisms which when administered in adequate amounts confer health benefits to the host.” Probiotics such as lactic acid bacteria are known to have antimutagenic [2], anticarcinogenic [3], and antidiarrheal [4] properties besides stimulating the immune system [5, 6] and improving infectious disease resistance [7] and inflammatory gastrointestinal [8]. They help in maintenance of balanced microbiota, improving lactose metabolism [9], and reducing blood pressure and cholesterol [10, 11]. Nevertheless, scientific evidence indicating that inactivated microbes positively affect human health can also be found in the literature [12]. Accordingly, products intentionally containing nonviable microbial cells are already present in the market (e.g., Lactéol Fort from PUMC Pharmaceutical Co., Ltd., and Fermenti Lattici Tindalizzati from Frau, AF United S.p.a.) [13].

The recent widespread use of lactic acid bacteria and bifidobacteria as probiotics can be attributed to scientific evidence that describes their beneficial effects on human health through the modulation of immune system activity [14], although the mechanisms involved in this immune modulation are not yet fully understood. Some of these mechanisms could include altering the balance of cytokines and interacting with cells of the immune system such as phagocytic mononuclear cells (monocytes and macrophages), polymorphonuclear leukocytes (neutrophils), and NK cells, as well as B and T lymphocytes [15].

Maassen et al. [16] showed that the synthesis of cytokines by the intestinal mucosa depends on the strain of* Lactobacillus* present. They emphasised the need to perform a careful selection of probiotic strain candidates. The benefits, effects, and mechanisms of action of probiotics in a host are yet to be fully elucidated.

Some known probiotic species, such as* Lactobacillus rhamnosus*,* L*.* acidophilus*, and* L*.* plantarum*, are used in researches that aim to clarify their benefits to the host [17–21]. Among these species,* Lactobacillus rhamnosus* is one of the most commonly used therapeutic probiotics. In some recent findings,* L*.* rhamnosus* GG showed significant reduction of the incidence of respiratory infections and the duration of diarrhea and improved the symptoms of atopic dermatitis [22]. Besides,* L*.* rhamnosus* GG inhibited the toxic effects of* Staphylococcus aureus* on epidermal keratinocytes [23].* L*.* rhamnosus* M21 activated humoral as well as cellular immune responses, conferring increased resistance to the host against a viral infection [24] and strain of* L*.* rhamnosus* ATCC 7469 ameliorated the enterotoxigenic* Escherichia coli*-induced diarrhea in piglets [25].* L*.* rhamnosus* L34 may produce factors capable of modulating inflammation stimulated by* Clostridium difficile* [26].

However, many of these beneficial effects are difficult to explain without first understanding the mechanisms responsible for the interaction between* Lactobacillus*, their secreted products, and host cells. Taking it into consideration, this research aimed to verify the capacity of the probiotic bacteria* L*.* rhamnosus* and their products to induce the synthesis of different cytokines by macrophages* in vitro*.

## 2. Materials and Methods

### 2.1. Preparation of* Lactobacillus* Suspensions

A standard strain of* L*.* rhamnosus* (ATCC 7469) was grown in Man-Rogosa-Sharpe (MRS, Oxoid, Basingstoke, Hampshire, England) agar and incubated at 37°C with 5% CO_2_ for 24 h, followed by incubation in MRS broth under the same conditions for 24 h. Three different suspensions of* L*.* rhamnosus* were then prepared:

1. Live* L*.* rhamnosus* (LLR) suspension: the culture was centrifuged for 10 min at 5000 rpm, the supernatants were discharged, and the pellet was suspended in sterile saline. This procedure was repeated two more times. During the last centrifugation, the pellet was suspended in apyrogenic sterile saline at a concentration of 5 × 10^7^ UFC/mL [27].
2. Heat-killed* L*.* rhamnosus* (HKLR) suspension: the live* L*.* rhamnosus* (LLR) suspension was autoclaved at 121°C for 15 min and centrifuged for 10 min at 5000 rpm, and the supernatant was removed and stored. The pellet was suspended in apyrogenic sterile saline.
3. Supernatant of heat-killed* L*.* rhamnosus* (SHKLR) suspension: supernatant was removed and stored of heat-killed* L*.* rhamnosus* (HKLR).

### 2.2. Cell Culture

The RAW 264.7 cell line (APABCAM, Rio de Janeiro, Brazil) was cultured in Dulbecco's modified Eagle's complete medium (DMEM, LGC Biotechnology, Cotia, Brazil), supplemented with 10% fetal bovine serum (FBS, Invitrogen, NY, USA) and 20 *μ*g/mL gentamicin, and incubated for 7 days, with medium culture exchange every 2 days, in a humidified atmosphere at 37°C with 5% CO_2_. The cells were grown to confluence in 75 cc tissue culture flasks prior to harvesting by scraping using a rubber spatula [27]. Viable cell counts were performed using the method of exclusion with trypan blue (0.5%, Sigma-Aldrich, St. Louis, MO, USA), 10^6^ cells were distributed onto 24-well microplates, and the medium volume was adjusted to 1 mL. The plates were incubated for 18 h (37°C/5% CO_2_) to permit cellular adherence prior to experimentation [27]. The supernatant was removed, and the adhered cells were washed twice with apyrogenic sterile saline (NaCl 0.85%). Afterward, 500 *μ*L of fresh DMEM supplemented with 10% fetal bovine serum was added without antibiotics for the culture with live bacteria and with antibiotics (20 *μ*g/mL gentamicin) for the other cultures [27].

### 2.3. Exposure of Cultures with* L*.* rhamnosus* Suspensions

Was added to the wells of the microplates with macrophages 500 *μ*L of each* L*.* rhamnosus* suspension, bringing the volume of each well to a total of 1 mL. The cells were incubated for 2.5 h at 37°C with 5% CO_2_ [27]. The supernatant was then removed, and the cells were washed twice with apyrogenic sterile saline (NaCl 0.85%). Following this, 1 mL of fresh DMEM supplemented with 10% fetal bovine serum with antibiotic was added, and the cells were incubated for 16 h at 37°C (5% CO_2_) [25]. The supernatants were then frozen and stored (at −80°C for approximately 3-4 weeks) prior to subsequent cytokine (TNF-*α*, IL-1*β*, IL-4, IL-6, IL-10, and IL-12), as described below.

The tests were performed in triplicate, 4 repetitions per group, for a total of 12 samples of each group (groups: LLR; HKLR; SHKLR; LPS; and negative control). The levels of cytokines generated by exposure of RAW 264.7 cells to* L*.* rhamnosus* were compared with those observed in RAW 264.7 cells that were cultured for the same duration with apyrogenic sterile saline (negative control) or LPS of* Escherichia coli* (10 EU/mL, positive control).

### 2.4. Quantification of Cytokine Levels

Cytokine levels (TNF-*α*, IL-1*β*, IL-4, IL-6, IL-10, and IL-12) were quantified using an enzyme-linked immunosorbent assay (ELISA). The DuoSet ELISA detection kit (R&D Systems, Minneapolis, MN, USA) was used according to the manufacturer's instructions. In all cases, detection antibody binding was visualized using the streptavidin-horseradish peroxidase conjugate and TMB (trimethylbenzidine) substrate system at an OD of 450 nm. After determining optical densities, cytokine levels (TNF-*α*, IL-1*β*, IL-4, IL-6, IL-10, and IL-12, pg/mL) in the macrophage culture supernatants were calculated using the GraphPad Prism 5.0 program. Results were analysed statistically using ANOVA and significant differences among means were determined by using Tukey's multiple-range test at *P* ≤ 0.05.

## 3. Results

**Figure 1:**
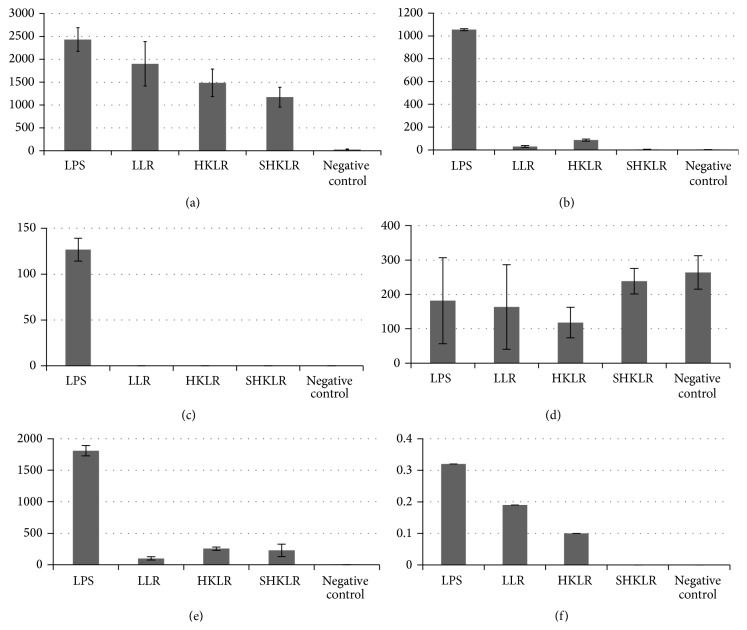
Cytokines secreted by macrophages after exposure to different suspensions: LPS (positive control); negative control (saline solution); LLR: live* L*.* rhamnosus*; HKLR: heat-killed* L*.* rhamnosus*; and SHKLR: supernatant of heat-killed* L*.* rhamnosus*. Mean values (pg/mL) ± standard deviation of (a) TNF-*α*, (b) IL-6, (c) IL-1*β*, (d) IL-12, (e) IL-10, and (f) IL-4.

The suspensions containing live* L*.* rhamnosus* (LLR) or heat-killed* L*.* rhamnosus* (HKLR) were able to induce significant production of TNF-*α* in the same amounts as LPS (*P* > 0.05). The suspensions with only the products of the microorganism (SHKLR) also significantly induced the production of this cytokine when compared with the negative control, although at a lower level than the other groups (LLR, HKLR, and LPS) (*P* < 0.05) (Figure 1(a)).

Stimulation with LPS (positive control) induces higher IL-6 production compared to the other groups (*P* < 0.05). The suspensions containing live* L*.* rhamnosus* (LLR) or heat-killed* L*.* rhamnosus* (HKLR) induced statistically similar IL-6 and this induced significantly higher production to SHKLR groups and negative control group (*P* < 0.05) (Figure 1(b)).

The secretion of IL-1*β* was not detected after the addition of any of the* L*.* rhamnosus* suspensions. Only stimulation with LPS produced detectable levels (Figure 1(c)).

Regarding IL-12, production was almost statistically similar in all groups evaluated (LLR, SHKLR, LPS, and negative control) (*P* > 0.05). The group stimulated with heat-killed* Lactobacillus rhamnosus* (HKLR) was the one who differed, with IL-12 levels being statistically lower than the other groups (*P* < 0.05) (Figure 1(d)).

The stimulation of IL-10 production with LPS induced greater production of the cytokines, and this amount was significantly different from the other groups (*P* < 0.05). The cultures stimulated with LLR, HKLR, and SHKLR also produced significant levels of IL-10 compared with the negative control group (*P* < 0.05). The HKLR and SHKLR groups were similar to each other (*P* > 0.05) and different from the LLR group (*P* < 0.05) (Figure 1(e)).

The production of IL-4 was detected in only some of the samples of LPS, LLR, and HKLR; however these values were not statistically significant so that all groups (LPS, LLR, HKLR, SHKLR, and negative control) were similar (*P* > 0.05) (Figure 1(f)).

## 4. Discussion

There are many bacteria with probiotic properties that can present different mechanisms of action, thus inducing different biological and clinical effects on the host [28]. It is always important to highlight the genus, species, and strain to precisely prescribe a probiotic product. In the present study, the standard strain ATCC 7469 of* Lactobacillus rhamnosus* was used because this strain has been frequently studied for its potential abilities to prevent and treat diseases such as herpes virus type 1, asthma, rheumatoid arthritis, dermatitis, and diarrhea [25, 29–32]. The* Lactobacillus* genus has also been observed to have important immunomodulatory effects against different pathogens [9, 33]; however, the exact mechanism of action and the best conditions to promote these benefits are not yet defined.

In the present research, macrophages were challenged with* L*.* rhamnosus*, and different results in cytokine production were observed. Living or dead* L*.* rhamnosus*, as well as their products alone, were able to induce the synthesis of TNF-*α*, and the suspensions containing live and dead cells of the microorganism generated the same amount of TNF-*α* as LPS. Other studies have found similar results in macrophage cultures using different probiotic strains [27, 34–36]. Khani et al. [30] also observed that live* L*.* rhamnosus* induced higher levels of TNF-*α*, suggesting that the entire bacteria promoted phagocytosis and consequently increased macrophage activation.

Live or dead* L*.* rhamnosus* generated significant levels of IL-6, but these levels were lower than with LPS. Habil et al. [37] observed that* L*.* rhamnosus*,* Lactobacillus fermentum*,* Lactobacillus plantarum*,* Lactobacillus salivarius*, and* Bifidobacterium breve* suppressed the production of IL-6 by macrophages that were primarily stimulated with LPS, showing that probiotics can activate or inhibit cytokine production depending on the conditions. It appears likely that probiotics can moderately stimulate the synthesis of proinflammatory cytokines in the instance of absence of inflammatory response and suppress it in situations of excessive response, which is a remarkable point in this study, once the macrophages had no stimuli with any pathogen.

The supernatant of* L*.* rhamnosus* did not induce IL-6 synthesis, thus suggesting the necessity of cell wall components for this event. According to Habil et al. [37], the manner in which the probiotic is introduced to the macrophage can affect cytokine production, that is, whether the products are associated with the wall (contact signal) or released as a soluble product (no contact signals).

In the present study, none of the* L*.* rhamnosus* suspensions induced detectable levels of IL-1*β*. Bleau et al. [38] also reported low levels of IL-1*β* in macrophages that were stimulated with different extracts of* Lactobacillus* in cultures and that LPS and live and heat-killed probiotics showed almost the same cytokine level production. Dong et al. [34, 39] observed an increase of this cytokine, however their study focused on mononuclear peripheral blood cells, and they stimulated these cells with different proportions of different probiotic bacteria for longer periods.

Detectable and apparently high levels of IL-12 were observed in all groups, including the negative control group that had the highest mean value, similar to groups stimulated with LPS and live* Lactobacillus* and supernatant of* Lactobacillus*. The group stimulated with dead* Lactobacillus* was the only one to show IL-12 lower levels. Other studies also reported high levels of IL-12 produced by macrophages or mononuclear blood cells that were challenged with probiotic strains [27, 34, 36]. However it appears that IL-12 production can be inhibited by other Gram-positive bacteria, as well as cell compounds such as peptidoglycan. A suspension with dead* L*.* rhamnosus* induced lower IL-12 production likely because of higher concentrations of cell wall compounds due to bacteria lysis from autoclaving. Therefore, probiotic bacteria can modulate macrophage function and suppress or increase IL-12 release [37, 40–42] and some cellular components can revert the cytokine profile induced by* Lactobacillus*, changing, for example, a profile of predominant IL-12 production to a profile of predominant IL-10 production, considered a suppressive cytokine IL-12.

Only a few samples in the study produced detectable levels of IL-4, making it difficult to discuss the effects of probiotics on the secretion of this cytokine. The literature has also reported controversial effects. Amital et al. [43] reported that lots of* Lactobacillus* strains show inhibitory effect on the release of IL-4 but, on the other hand, they are potent stimulators of IFN-*γ*, IL-12, and TNF-*α*, corroborating our results. However, Drago et al. [44] observed that the strains of* L*.* salivarius* (LDR0723 and CRL1528) promoted a significant increase in IL-12 and IFN-*γ* and a reduction of IL-4 and IL-5, while the strains BNL1059 and RGS1746 increased the Th2 response. Drago and coworkers concluded that the modulation response by* L*.* salivarius* was strain dependent, meaning that different strains of the same species can produce different cytokines.

The current study shows that* L*.* rhamnosus*, as well as their products alone, were able to induce the synthesis of proinflammatory cytokines and possibly a response profile of Th1 type, which is important in the defense against intracellular pathogens most and contrary to hypersensitivity frames, which are usually caused by imbalance in Th2 anti-inflammatory response.* Lactobacillus* were also able to induce IL-10 production, which has the regulatory functions and can inhibit Th1 response and when excessive can lead to tissue damage. Therefore there must be a balance between these responses (Th1/Th2), and these bacteria,* L*.* rhamnosus*, seem to have potential role in the modulation as well as the maintenance of the immune system balance. Rajput et al. [45] also showed that the administration of the probiotic strains of* Saccharomyces boulardii* and* Bacillus subtilis* B10 was able to increase the production of IL-10 in chickens.

However, it is important to note that this is an* in vitro* study. We used only one lineage of resting cells, because they had not been subjected to any treatment or early stimulation. Our experimental conditions were different from* in vivo* conditions, where many cells are present. Most importantly, our experiment did not take into consideration the effects of lymphocytes, which are the primary cells in cytokine production and in the organization of adaptive responses.* In vivo* situations present a more complex system in which other stimuli such as pathogens act and interact.

Live and heat-killed* L*.* rhamnosus* suspensions were able to induce the synthesis of different cytokines with proinflammatory (TNF-*α* and IL-6) or regulatory (IL-10) functions, suggesting that* L*.* rhamnosus* ATCC 7469 is capable of exerting immunoregulatory effect on macrophages.

## References

[1] WHO-World Health Organization Food and agriculture organization of the United Nations. Evaluation of health and nutritional porperties of probiotics in food including powder milk with live lactic and bacteria. http://www.who.int/foodsafety/publications/fs_management/probiotics/en/index.html.

[2] LeBlanc A. M., Matar C., LeBlanc N., Perdigón G. (2005). Effects of milk fermented by *Lactobacillus helveticus* R389 on a murine breast cancer model.. *Breast Cancer Research*.

[3] Wollowski I., Rechkemmer G., Pool-Zobel B. L. (2001). Protective role of probiotics and prebiotics in colon cancer. *The American Journal of Clinical Nutrition*.

[4] Clancy R. (2003). Immunobiotics and the probiotic evolution. *FEMS Immunology and Medical Microbiology*.

[5] Van Hai N., Buller N., Fotedar R. (2009). The use of customised probiotics in the cultivation of western king prawns (*Penaeus latisulcatus* Kishinouye, 1896). *Fish and Shellfish Immunology*.

[6] Wickens K., Black P. N., Stanley T. V. (2008). A differential effect of 2 probiotics in the prevention of eczema and atopy: a double-blind, randomized, placebo-controlled trial. *Journal of Allergy and Clinical Immunology*.

[7] Li J., Tan B., Mai K. (2008). Immune responses and resistance against *Vibrio parahaemolyticus* induced by probiotic bacterium *Arthrobacter* XE-7 in Pacific white shrimp, *Litopenaeus vannamei*. *Journal of the World Aquaculture Society*.

[8] Ryan K. A., O'Hara A. M., Van Pijkeren J.-P., Douillard F. P., O'Toole P. W. (2009). Lactobacillus salivarius modulates cytokine induction and virulence factor gene expression in *Helicobacter pylori*. *Journal of Medical Microbiology*.

[9] Ohashi Y., Ushida K. (2009). Health-beneficial effects of probiotics: its mode of action. *Animal Science Journal*.

[10] Hlivak P., Odraska J., Ferencik M., Ebringer L., Jahnova E., Mikes Z. (2005). One-year application of probiotic strain *Enterococcus faecium* M-74 decreases serum cholesterol levels. *Bratislavské Lekárske Listy*.

[11] Shieh M.-J., Shang H.-F., Liao F.-H., Zhu J.-S., Chien Y.-W. (2011). *Lactobacillus fermentum* improved intestinal bacteria flora by reducing *Clostridium perfringens*. *e-SPEN, the European e-Journal of Clinical Nutrition and Metabolism*.

[12] Kataria J., Li N., Wynn J. L., Neu J. (2009). Probiotic microbes: do they need to be alive to be beneficial?. *Nutrition Reviews*.

[13] Taverniti V., Guglielmetti S. (2011). The immunomodulatory properties of probiotic microorganisms beyond their viability (ghost probiotics: proposal of paraprobiotic concept). *Genes and Nutrition*.

[14] Yasuda E., Serata M., Sako T. (2008). Suppressive effect on activation of macrophages by *Lactobacillus casei* strain shirota genes determining the synthesis of cell wall-associated polysaccharides. *Applied and Environmental Microbiology*.

[15] Nayak S. K. (2010). Probiotics and immunity: a fish perspective. *Fish & Shellfish Immunology*.

[16] Maassen C. B. M., van Holten-Neelen C., Balk F. (2000). Strain-dependent induction of cytokine profiles in the gut by orally administered *Lactobacillus* strains. *Vaccine*.

[17] Liu C.-F., Tseng K.-C., Chiang S.-S., Lee B.-H., Hsu W.-H., Pan T.-M. (2011). Immunomodulatory and antioxidant potential of *Lactobacillus exopolysaccharides*. *Journal of the Science of Food and Agriculture*.

[18] Matsubara V. H., Silva E. G., Paula C. R., Ishikawa K. H., Nakamae A. E. M. (2012). Treatment with probiotics in experimental oral colonization by *Candida albicans* in murine model (DBA/2). *Oral Diseases*.

[19] Villena J., Chiba E., Tomosada Y. (2012). Orally administered *Lactobacillus rhamnosus* modulates the respiratory immune response triggered by the viral pathogen-associated molecular pattern poly(I:C). *BMC Immunology*.

[20] Yang H.-J., Min T. K., Lee H. W., Pyun B. Y. (2014). Efficacy of probiotic therapy on atopic dermatitis in children: a randomized, double-blind, placebo-controlled trial. *Allergy, Asthma and Immunology Research*.

[21] Yun B., Oh S., Griffiths M. W. (2014). *Lactobacillus acidophilus* modulates the virulence of *Clostridium difficile*. *Journal of Dairy Science*.

[22] Ciszek-Lenda M., Nowak B., Śróttek M., Gamian A., Marcinkiewicz J. (2011). Immunoregulatory potential of exopolysaccharide from *Lactobacillus rhamnosus* KL37: effects on the production of inflammatory mediators by mouse macrophages. *International Journal of Experimental Pathology*.

[23] Mohammedsaeed W., McBain A. J., Cruickshank S. M., O'Neill C. A. (2014). *Lactobacillus rhamnosus* GG inhibits the toxic effects of *Staphylococcus aureus* on epidermal keratinocytes. *Applied and Environmental Microbiology*.

[24] Song J. A., Kim H. J., Hong S. K. (2014). Oral intake of *Lactobacillus rhamnosus* M21 enhances the survival rate of mice lethally infected with influenza virus. *Journal of Microbiology, Immunology and Infection*.

[25] Li X.-Q., Zhu Y.-H., Zhang H.-F. (2012). Risks associated with high-dose lactobacillus rhamnosus in an *Escherichia coli* model of piglet diarrhoea: intestinal microbiota and immune imbalances. *PLoS ONE*.

[26] Boonma P., Spinler J. K., Venable S. F., Versalovic J., Tumwasorn S. (2014). Lactobacillus rhamnosus L34 and *Lactobacillus casei* L39 suppress *Clostridium difficile*-induced IL-8 production by colonic epithelial cells. *BMC Microbiology*.

[27] Cross M. L., Ganner A., Teilab D., Fray L. M. (2004). Patterns of cytokine induction by gram-positive and gram-negative probiotic bacteria. *FEMS Immunology and Medical Microbiology*.

[28] Francavilla R., Fontana C., Cristofori F. (2012). Letter: identication of probiotics by specific strain name. *Alimentary Pharmacology & Therapeutics*.

[29] Jang S.-O., Kim H.-J., Kim Y.-J. (2012). Asthma prevention by *Lactobacillus rhamnosus* in a mouse model is associated with CD4^+^CD25^+^Foxp3^+^T cells. *Allergy, Asthma and Immunology Research*.

[30] Khani S., Motamedifar M., Golmoghaddam H., Hosseini H. M., Hashemizadeh Z. (2012). In vitro study of the effect of a probiotic bacterium *Lactobacillus rhamnosus* against herpes simplex virus type 1. *Brazilian Journal of Infectious Diseases*.

[31] Marsella R., Santoro D., Ahrens K. (2012). Early exposure to probiotics in a canine model of atopic dermatitis has long-term clinical and immunological effects. *Veterinary Immunology and Immunopathology*.

[32] Nowak B., Ciszek-Lenda M., Śróttek M. (2012). *Lactobacillus rhamnosus* exopolysaccharide ameliorates arthritis induced by the systemic injection of collagen and lipopolysaccharide in DBA/1 mice. *Archivum Immunologiae et Therapiae Experimentalis*.

[33] Zhu Y.-H., Li X.-Q., Zhang W., Zhou D., Liu H.-Y., Wang J.-F. (2014). Dose-dependent effects of *Lactobacillus rhamnosus* on serum interleukin-17 production and intestinal T-cell responses in pigs challenged with *Escherichia coli*. *Applied and Environmental Microbiology*.

[34] Dong H., Rowland I., Tuohy K. M., Thomas L. V., Yaqoob P. (2010). Selective effects of *Lactobacillus casei* Shirota on T cell activation, natural killer cell activity and cytokine production. *Clinical and Experimental Immunology*.

[35] Perdigon G., Galeano C. M., Valdez J. C., Medici M. (2002). Interaction of lactic acid bacteria with the gut immune system. *European Journal of Clinical Nutrition*.

[36] Won T. J., Kim B., Song D. S. (2011). Modulation of Th1/Th2 balance by *Lactobacillus* strains isolated from Kimchi via stimulation of macrophage cell line J774A.1 in vitro. *Journal of Food Science*.

[37] Habil N., Al-Murrani W., Beal J., Foey A. D. (2011). Probiotic bacterial strains differentially modulate macrophage cytokine production in a strain-dependent and cell subset-specific manner. *Beneficial Microbes*.

[38] Bleau C., Savard R., Lamontagne L. (2007). Murine immunomodulation of IL-10 and IL-12 induced by new isolates from avian type 2 *Lactobacillus acidophilus*. *Canadian Journal of Microbiology*.

[39] Dong H., Rowland I., Yaqoob P. (2012). Comparative effects of six probiotic strains on immune function in vitro. *British Journal of Nutrition*.

[40] Foligne B., Nutten S., Grangette C. (2007). Correlation between in vitro and in vivo immunomodulatory properties of lactic acid bacteria. *World Journal of Gastroenterology*.

[41] Ichikawa S., Fujii R., Fujiwara D. (2007). MyD88 but not TLR2, 4 or 9 is essential for IL-12 induction by lactic acid bacteria. *Bioscience, Biotechnology and Biochemistry*.

[42] Shida K., Suzuki T., Kiyoshima-Shibata J., Shimada S.-I., Nanno M. (2006). Essential roles of monocytes in stimulating human peripheral blood mononuclear cells with *Lactobacillus casei* to produce cytokines and augment natural killer cell activity. *Clinical and Vaccine Immunology*.

[43] Amital H., Gilburd B., Shoenfeld Y. (2007). Probiotic supplementation with *Lactobacillus casei* (Actimel) induces a Th1 response in an animal model of antiphospholipid syndrome. *Annals of the New York Academy of Sciences*.

[44] Drago L., Nicola L., Iemoli E., Banfi G., De Vecchi E. (2010). Strain-dependent release of cytokines modulated by *Lactobacillus salivarius* human isolates in an *in vitro* model. *BMC Research Notes*.

[45] Rajput I. R., Li L. Y., Xin X. (2013). Effect of *Saccharomyces boulardii* and *Bacillus subtilis* B10 on intestinal ultrastructure modulation and mucosal immunity development mechanism in broiler chickens. *Poultry Science*.

